# Baby-friendly workplace initiatives in child feeding practice as predictors of infant and young child anthropometric indices in public health facilities of Southern Ethiopia

**DOI:** 10.1186/s40795-024-00862-w

**Published:** 2024-04-02

**Authors:** Habtamu Hasen, Samuel Kusheta, Efrata Abuhay, Hamdela Tumiso, Yenatfanta Leuel, Dinku Daniel, Mesganew Amare, Abraham Samuel, Yitagesu Habtu

**Affiliations:** 1 Department of Public Health, Hossana College of Health Science, Hossana, Ethiopia; 2 Department of midwifery, Hossana College of Health Science, Hossana, Ethiopia; 3 School of midwifery, Faculty of Health Science, Institute of Health, Jimma University, Jimma, Ethiopia; 4 Department of emergency health service, Hossana College of Health Science, Hossana, Ethiopia

**Keywords:** Baby-friendly workplace, Children, Ethiopia, Nutrition status

## Abstract

**Background:**

Baby-friendly workplace is an effective evidence based initiative developed by the World Health Organization to protect and support maternal knowledge, beliefs, and confidence in infant and young child feeding practices. However, studies that show the effect of the baby-friendly workplace initiative on the nutritional status of infant and young children are not available in Ethiopia. Therefore, this study aimed to assess the nutritional status among baby friendly initiatives service utlizers and non utlizers children age 6–24 months in public health facilities of Southern Ethiopia.

**Methods:**

We conducted a comparative cross-sectional study from 1 to 30 June 2022 among 220 mothers with children aged 6–24 months. Data were collected through face-to-face interviews using a structured questionnaire. Data were entered into Epidata Software version 4.2 and then exported to IBM SPSS version 26 software for analysis. Chi-square and Fisher exact test were used to assess the differences between users and non-users of the baby friendly workplace initiative. Logistic regression model was used to determine the association between dependent and independent variables. Adjusted odds ratio (AOR) with a 95% confidence interval was computed. *P*-values < 0.05 at a 95% confidence level were considered statistically significant.

**Result:**

The mean (SD) scores of weight for age (WAZ), height for age (HAZ), and weight for height (WHZ) were − 0.38 (1.34),-0.17(2.62) and-0.35 (1.84) respectively. After adjusting for covariates, children aged 6–24 months who did not use baby friendly workplace initiatives were 2.26 times more likely to have stunting compared to the users of baby friendly workplace initiative (AOR 2.26, 95% CI: 1.05, 4.88). However, both wasting (AOR: 0.42; 95% CI:0.13, 1.37) and underweight (AOR: 1.09; 95% CI: 0.45, 2.60) were not significantly associated with the use of baby friendly workplace initiatives.

**Conclusion:**

The use of baby friendly work place initiatives was successful in improving nutritional status, specifically chronic malnutrition in children. Strengthening and scaling up the baby friendly work place initiative program has the potential to reduce chronic malnutrition in Ethiopia and other similar settings with high burden of malnutrition areas, by implementing it in public facilities.

## Background

Globally in 2020, an estimated 149 million children under-five were stunted, while 45 million were wasted, and 38.9 million were overweight or obese [1]. Sub-Saharan Africa including Ethiopia have the highest of undernutrition among children under the age of five [1].

Appropriate infant and young child feeding (IYCF) is critical for the optimal growth and development of children particularly during the first 2 years of life [2–5]. Optimal nutrition during this window period in children lowers morbidity and mortality by reducing the risk of chronic diseases, and fostering better overall development in later life. It also helps to avert the damage caused by malnutrition which ultimately leads to significant social, political, and economic losses [1, 6].

Globally, the implementation of the baby-friendly workplace initiative (BFWI), otherwise known as the Baby-friendly Hospital Initiative (BFHI) is one of the many evidence-based interventions that may be adapted to different country contexts to implement practices that protect, promote and support appropriate IYCF practices including breastfeeding.This is achieved by improving maternal knowledge, beliefs, and confidence in IYCF practices [7, 8].

In Ethiopia, inappropriate IYCF practices are still among the major challenges faced by employed mothers that need to addressed through a variety of interventions. Child malnutrition remains a persistent public health problem in the country. The Ethiopian mini Demographic Health Survey (EDHS) of 2019, for example, shows that 37% of children under the age 5 are stunted (short for their age); 7% are wasted (thin for their height); 21% are underweight; and 2% are overweight. Only 59% of infants under the age of 6 months are exclusively breastfed and 11% of children aged 6–23 months are fed a minimum acceptable diet [9].

One of the major determinants is mothers’ employment status,which may hinder the implementation of optimal breastfeeding. This is because of short duration of maternity leave, lack of flexible work time, and lack of lactation breaks, all of which act as the barriers to breastfeeding. Furthermore, public legislation in Ethiopia poorly protects the breastfeeding rights of most new mothers who are employed [10–14]. Such challenges could be prevented or reduced through implementing various of interventions, including the BFWI) also called the Mother-Friendly Workplace Initiative) which was launched in 1993 and revised in 2018 to promote combining women’s work and breastfeeding [15]. Prior evidences have shown that the use of the baby-friendly workplace initiative is successful in improving the rates of exclusive breastfeeding,complementary feeding practices, and the nutritional status of children [16–19].

The government of Ethiopia has also launched the ‘multi-sectoral National Nutrition Program II to improve the nutritional status of children [20]. However, the assessment of the status of infant and young child feeding (IYCF) practice, policy, and programs revealed that Ethiopia had very low implementation of the Baby-Friendly Hospital Initiative (BFHI) [21]. Despite some areas are implementing the initiative,the lack of implementation of the Baby-Friendly workplace Initiative and the absence of workplace breastfeeding laws in Ethiopia have fueled unresolved poor child-feeding practices [22–24].

In Southern Ethiopia, in the Silte Zone public health facilities, efforts have been made to implement baby friendly work place initiatives (BFWPI) by renaming them as “Onsite Workplace Childcare Center (OWCC)”. The aim of these initiatives is to improve exclusive breast feeding and complementary feeding practices, as well as nutritional status [25]. However, there is a lack of previous studies that show the effect of baby-friendly workplace initiative on the nutritional status of children specifically in Ethiopia and the study area of Southern Ethiopia. Therefore, the study aimed to assess the nutritional status of children age 6–24 months on baby friendly initiatives service utlizers and non utlizers in public health facilities of Southern Ethiopia.

The findings of this study will provides valuable information for the Ministry of Health and NGOs regarding the implementation and promotion of breastfeeding practices.

## Method and materials

### Study area, period, and design

We used a facility-based comparative cross-sectional study in Silte Zone from 1 to 30 June 2022.Silte Zone is found in Southern Nations Nationalities and Peoples’ Region (SNNPR) of Ethiopia. Silte Zone is located 172 km away from Addis Ababa, the capital city of Ethiopia in the southwest direction and 107 km from Hawassa, the capital city of SNNPR. This zone consists of 5 administration towns, 10 rural woreda, and 212 kebeles. Based on the last Census conducted by the Central Statistical Agency of Ethiopia (CSA), in 2018 this Zone has an estimated population of 1,017,557. The Zone currently has 4 hospitals and 33 health centers. Around 13 health facilities have baby-friendly workplace initiatives.

### Study participants

All care givers with children aged 6–24 months working in public health facilities of Silte zone were a source population and selected caregivers with children aged 6–24 months working in public health facilities of Silte zone considered as study population. Care givers with pair of children aged 6–24 months were selected as the respondents of the study.

Accordingly, the study participants were categorized into two groups: mothers with children aged 6–24 months who were using of baby-friendly workplace initiatives program and other groups were mothers with children aged 6–24 months who were not using a baby-friendly workplace initiatives program. Mothers with children aged 6–24 months who reside in the study area and working in public health facilities were included in the study. All children with evidence of physical impairment, or seriously ill were excluded.

### Sample size determination and Sampling Procedure

The sample size was calculated by applying two population proportion formulas using Epi-Info version 7 by taking type one error of 5%, 80% power, a design effect of 1.5, and a 1:1 ratio of an exposed group (mothers with children aged 6–24 months who were using BFWPI to a non-exposed group (mothers with children aged 6–24 months who were not using BFWPI )(*r* = 1). Assuming the proportion of wasting among children aged 6–11 months (25.4%) and (AOR = 2.78) from a study conducted in Gurage Zone [26]. Considering 5% non-response rate, 110 mothers with children aged 6–24 months who were using BFWPI and 110 mothers with children aged 6–24 months who did not use BFWPI was included.

Multi-stage stratified sampling was used to collect data from respondents across the zone and stratified by rural and urban Woredas. Second, five of the fifteen Woredas of Silte Zone (Kibet town, Werabe town, Silti Woreda, Alicho Woreda, and eastern Azernet Berbere Woreda) were randomly selected. We prepared lists of all the public health facilities that start the initiatives in the Zone. Due to the small sample size of children aged 6–24 months who utilized BFWI (*n* = 110 children), we took all study units. Then, one-third of health facilities (health centers and hospitals) from Woredas and towns were selected, followed by a random selection of caregivers with children aged 6–24 months using the health facilities’ registry as a frame. The sample size for non-utilizer of BFWI was allocated based on proportional size allocation methods.

### Variables and measurements

Outcome variable of interest, nutritional status was determined based on anthropometric indices: Weight-forage Z-score (WAZ), Height-for-age Z-score (HAZ), and weight-for-height Z-score (WHZ). We used the World Health Organization Child Growth Standards to classify nutritional status [27, 28]. Accordingly, children whose weight-for-age z scores, height-for-age z scores, and weight-for-height Z scores were less than − 2 SDs below the median for their age and gender were defined as underweight stunting and wasting, respectively [27, 28]. Optimal achievement of minimum dietary diversity was defined as proportion of children with 6–24 months of age who received foods from four or more food groups of the seven food groups [29]. Length and weight measurements were taken in duplicate using calibrated equipment and standardized techniques. Length (height) was measured in the recumbent position to the nearest 0.1 cm using a measuring board with an upright base and movable headpiece made by Seca, Germany. Weight was measured using weighing scales (Seca, Germany) (+ 10 g precision) with light clothing. The independent variables include socio-demographic information of mother with children, dietary intake, complementary feeding and breast feeding information and facility related factors which were collected through face-to-face interviews by trained interviewer.

### Data management and analysis

The data were checked for inconsistencies and completeness before data entry. Then, data were entered using Epi data Software version 4.2 and analyzed by IBM SPSS version 26. We used the WHO AnthroPlus Software to convert nutritional data from anthropometric measurement into Z-score of the indices. Descriptive statistical analysis was carried out to identify frequency, percentage, and mean for continuous variables. Pearson’s chi-squared test was used to assess differences between groups (those mothers with children aged 6–24 months who were using BFWPI and those mothers with children aged 6–24 months who were not using BFWPI ) based on their socio-demographic characteristics.Binary logistic regression analysis was used to ascertain the association between the dependent and independent variables. Variables with a significant association at *P* < 0.25 in the binary analysis were entered into a multivariable logistic regression model. The goodness of fit of the model was checked using the Hosmer Lemeshow test of goodness of fit.

### Data quality assurance

The questionnaires were translated to Amharic and then back translated to English to assure the quality of data. Four-day training for data collectors and supervisors was given. Standardization exercise was made for taking of anthropometric measurements and using of experienced data collectors in data collection process.

## Results

### Basic characteristics of the study participants

Table 1 summarizes the characteristics of the users of the BFWP initiative and non-users. Overall, more than half (51.9%) of the study participants were females. The mean (± SD) age of the children was 15.0 ± 4.69 months. Of the study participants, 198 (95.2%) were urban residents. Nine in ten mothers (users and non-users of BFWP initiatives) had college and above educational status. Eighty-one (76.4%) users of BFWPI and 77 (75.5%) non-users of BFWPI ranged from 25 to 34 years old and the majority of both groups were married.

**Table 1:** Basic characteristics of the study participants by using BFWPI status in Southern Ethiopia, 2022

Variables	BFWP initiative users n (%)	BFWP initiative non-users n (%)	Total N (%)	P^a^
Residence				0.174
Urban Rural	95 (93.1) 7 (6.9)	103 (97.2) 3 (2.8)	198 (95.2) 10 (4.8)	
Educational status of mother				
Secondary and lower school College and above	21 (10.1) 187 (89.9)	10 (9.8) 92 (90.2)	11 (10.4) 95 (89.6)	0.891
Maternal age (year)				0.810
≥ 24 25–34 ≥ 35	6(5.9) 77 (75.5) 19 (18.6)	8 (7.5) 81(76.4) 17(16.0)	14(6.7) 158 (76.0) 36 (17.3)	
Marital status				0.807
Single Married	5 (4.9) 97 (95.1)	6 (5.7) 100 (94.3)	11 (5.3) 197 (94.7)	
Family size				0.212
≤ 5 > 5	90 (88.2) 12 (11.8)	87 (82.1) 19 (17.9)	177 (85.1) 31 (14.9)	
Health profession				0.375
Yes No	75 (73.5) 27 (26.5)	72 (67.9) 34 (32.1)	147 (70.7) 61 (29.3)	
Workload				0.208
Yes No	70 (68.6) 32 (31.4)	81 (76.4) 25 (23.6)	151 (72.6) 57 (27.4)	

^a^ Pearson’s chi-square and fisher exact test

Regarding to professional status, 67.9% of BFWPI users and 73.5% of BFWPI non-users were health professionals. Nearly three out of four users of BFWPI and 70(68.6%) of non-users of BFWPI had a perceived workload (Table 1). Out of 208 children, 71.6% of them were in the age range of 12–24 months and more than (51.9%) were female (Table 4).

### Child feeding practices

Of those children, 86.5% had optimal dietary diversity practices. An estimated 46 (22.1%) children had experienced diarrhoea in the last 4 weeks before the study period. The majority (96.2%) of children started complementary feeding practice. Only 7.5% of children’s mothers planned to breastfeed up to 24 months of age. Table 2 presents child feeding practices and basic demographic variables. The users of BFWPI did not significantly differ from non-users of BFWPI with regard to sex, age of children, birth order, optimal dietary diversity practice, starting complementary feeding, dietary diversity practice, and initiation to continue breastfeeding. However, these two groups were significantly different in diarrhoeal disease statuses in the last 4 weeks before the study period (Table 4).

**Table 2:** Anthropometric Z-scores of Infants and young Children in Southern Ethiopia, 2022

Z scores	Age groups	N	%<-3SD95% CI)	%<-2SD(95% CI)	Mean	SD
Weight-for-height	6–11	45	15.6 (3.9, 27.3)	24.4 (10.8, 38.1)	-1.04	1.96
	12–24	163	7.3 (2.8, 11.8)	22.9 (8.6, 28.1)	-0.13	1.81
	Total	208	8.7 (4.6, 12.7)	16.3 (11.1, 21.6)	-0.35	1.84
Height-for-age	6–11	45	0 (0, 1.1)	11.1(0.8, 21.4)	0.74	2.38
	12–24	163	22.2(8.1, 28.1)	25.2 (17.9, 32.4)	-0.36	2.71
	Total	208	10.6 (6.2, 15)	22.6(16.7, 28.5)	-0.17	2.62
Weight for age	6–11	45	2.2 (0, 7.6)	11.1(0.8, 21.4)	-0.52	1.36
	12–24	163	4 (0.5, 7.4)	18.2 (3.3, 28.1)	-0.72	1.36
	Total	208	3.4(0.7, 6.1)	8.7(4.6, 12.7)	-0.38	1.34

### Anthropometric Z-scores of infant and young children

The mean (SD) scores of weight for age (WAZ), height for age (HAZ), and weight for height (WHZ) were − 0.38 (1.34),-0.17(2.62) and-0.35 (1.84), respectively. The prevalence of wasting, stunting, and being underweight were 16.3%, 23.6%, and 8.7% respectively. The proportion of moderate wasting was (16.3%) while severe wasting was 8.7%. A severe form of stunting was 10.6% while moderate stunting was 22.6% Table 2 shows anthropometric Z-Scores of children (Table 2).

### Effect of BFWPI on anthropometric status of infant and young children

The multivariable logistic regression analysis showed that children who were not using BFWPI were 2·26 times more likely stunted than children who used BFWPI (AOR 2.26, 95%CI: 1.05.4.88) after being adjusted for sex, age, birth order, family size, maternal age, dietary diversity score and availability of materials for hand washing and feeding (Table 3). However, there was no statistically significant difference between the two groups with regard to wasting and underweight after adjusting for different variables. Only 15.7% of children were wasted in the users of BFWPI compared to 17% of non-users of BFWPI (*p* = 0.801) (Table 4).

**Table 3:** Effect of BFWPI on nutritional status of infant and young children Southern Ethiopia, 2022

Nutritional status	COR 95% CI	AOR 95% CI
Stunting	1.54(0.81,2.96)	2.26 (1.05.4.88)^a^
Underweight	0.45 (0.16, 1.25)	0.42 (0.13,1.37)^b^
Wasting	1.09 (0.52, 2.29)	1.09 0.45,2.60)^c^

a: Adjusted for family size, child age less than five years, maternal age, dietary diversity score, and availability of materials for hand washing and feeding

b:Adjusted for residence, past illness, educational status, husband’s occupation, profession, and service year, income, getting support from coordinators, colleagues, and provision of time for breastfeeding

c: Adjusted for variables like residency, complementary feeding, professional category, husband occupation, educational level of mother, dietary diversity score, children age, children sex, get support from your co -worker, provision of adequate time for breast feeding, availability of materials for cooking, storing, and availability of materials for hand and feeding

**Table 4:** Infant and young children feeding practices by using BFWPI in Southern Ethiopia, 2022

Variables	BFWP user n (%)	BFWP non-user n (%)	Total N (%)	P^a^
Sex				
Male Female	46 (45.1) 56 (54.9)	54 (50.9) 52 (49.1)	100 (48.1) 108 (51.9)	0.399
Age of children				0.743
6–11 month 12–24 month	30 (29.4) 72 (70.6)	29 (27.4) 77 (72.6)	59 (28.4) 149 (71.6)	
Birth order				0.238
First	42 (41.2)	33 (31.1)	75 (36.1)	0.238
Second	31 (30.4)	46 (43.4)	77 (37.0)	
Third	20(19.6)	17 (16.0)	37 (17.8)	
Fourth and above	9 (8.8)	10 (9.4)	19 (9.1)	
Optimal dietary diversity				0.913
≥4 food groups < 4 food groups	88 (86.3) 14 (13.7)	92 (86.8) 14 (13.2)	180 (86.5) 28 (13.5)	
Diarrhea in the last 4 weeks				0.006
Yes No	9 (8.8) 93 (91.2)	37 (34.9) 69 (65.1)	46 (22.1) 162 (77.9)	
Start complementary feeding				0.305
Yes No	100 (98.0) 2 (2.0)	100 (94.3) 6 (5.7)	200 (96.2) 8 (3.8)	
Intention to continue BF				0.431
≤ 24 months > 24 months	5 (4.9) 97 (95.1)	8 (7.5) 98 (92.5)	13 (6.3) 195 (93.7)	
Dietary diversity practice				0.213
Good Poor	11 (39.3) 50 (27.8)	17 (60.7) 130 (72.2)	28 (13.5) 180 (86.5)	

^a^ Pearson’s chi-square and fisher exact test result

Multivariable logistic regression analysis neither showed a significant effect of the BFWPI on wasting nor on underweight status of children aged 6–24 months.

In the multivariable logistic regression model, using BFWPI did not bring significant effect on wasting status when adjusted for potential confounders including socio-demographic and children characteristics (AOR;1.09,95% CI:0.45,2.60). Furthermore, using BFWPI did not have a significant effect on underweight status when adjusting for residence, past illness, educational status, husband’s occupation, profession, service year, income, getting support from coordinators, colleagues, and provision of time for breastfeeding (AOR = 0.42,95% CI: 0.13, 1.37) (Table 3).

## Discussion

Despite studies showing predictors of optimal infant and young child feeding practices and failures in the scores of anthropometric indices are available in Ethiopia including Southern Ethiopia, studies assessing the nutritional status of children age 6–24 months on baby friendly initiatives service utlizers and non utlizers in public health facilities of Ethiopia.

are almost non existance as to the researcher’s knowledge. Such evidences build the efforts of implementing and scaling up effective approaches to contextualized child feeding strategies. Especially, in the context of Ethiopia,where they are inequalities and equities in nutrition, poor socio-economic conditions, inadequate nutritional interventions due to other multiple competitive needs and cultural factors, updated information for action is required on new nutritional initiatives and programs to assess their effect on reducing the burden of malnutrition in children. Therefore, our study assessed whether baby-friendly workplace initiative brought significant differences in child nutrition status through comparing child anthropometric indices between study participants working in BFWPI implementing and non-implementing public health facilities.

Our study highlighted that children whose mothers had not used baby-friendly workplace initiatives were more stunted than their counterparts. Our finding is supported by a study conducted in the Eastern Mediterranean Region,which indicated that poor nutritional status was associated with low BFHI implementation and low exclusive breastfeeding rates [18]. Moreover, in support of the current findings on the potential use of BFWPI, studies from Kenya and India revealed that baby-friendly workplace initiatives increased the duration of exclusive breast feeding and complementary feeding practices [16, 30]. Moreover, the observed association between baby-friendly workplace initiatives and anthropometric indices in children was not consistent with the study conducted in Kenya [19]. This inconsistency could be attribute to the difference in the study participants between the Kenya study, which included all children aged up to 24 months and the current study, which included study participants with aged 6–24 months. Additionally, there is discrepancy difference in baby-friendly workplace support.For instance, in Kenya, baby-friendly workplace initiatives were supported by government and non-governmental organizations where as in Ethiopia,baby-friendly workplace initiatives were lack support by both sides and the initiatives were started by the willingness of local health facilities coordinators.

Studies conducted in North and South Carolina and Maryland,USA as well as in Norwegian, Lebanon, and Korea counties reported that the a significant association between rates of exclusive breastfeeding and duration of exclusive breastfeeding with the presence of baby friendly workplace initiatives [17, 31–34]. The association between breast feeding and nutritional status was explained in the study done in Kenya.The study indicated that There was a significant association between delay in time of breastfeeding initiation and stunting, discontinuation of breastfeeding and underweight [35]. Further study from India also support the statistically significant association between nutritional status and breast feeding and complementary feeding [36]. Additional supporting findings from the intervention for pregnant and lactating women and their infants in Cameroon with a Baby-Friendly space indicated that the program has a positive effect on maternal mental health, childcare practices, and early child development [37].

The present study also revealed that there was no statistically significant difference concerning wasting and underweight after adjusting for different variables. This lack of statistical significance is undoubtedly due to the small sample size of children aged 6–24 months (*n* = 208) which limited our power to detect differences between BFWP initiatives user groups (*n* = 102) and BFWP initiatives non-user groups (*n* = 106). Furthermore, the implementation of the full BFHI package is a complex intervention in which the lack of supplementary material for the preparation of food and limited financial support for caregivers in the baby waiting corners would contribute to the nature of WHZ scores, making them susceptible to acute changes in diet or health. Supporting evidences from a study conducted in Gambella refuge Camp, Ethiopia, on baby-friendly spaces, reported an improvement in anthropometric measures showed improvement. However, the percentage of children at risk of malnutrition, as measured by Weight-for-Age Z-scores (WAZ), increased from 6.5 to 8.8% [38].

The finding was in line with a study done in Kenya in which there was no statistically significant difference in wasting among children aged 0–24 months. However, our findings were not consistent with an age-stratified analysis of the Kenya study. It revealed that among children aged greater than 6 months,there was no statistically significant difference in stunting (*p* = 0.514 ), but there is statistically significant difference in underweight (*p* < 0.001), and wasting (*p* = 0.022) [19]. A more robust supporting findings from systematic review and meta-analysis did not identify intervention benefits for child cognitive and other growth outcomes [39].

However, precaution needed in the interpretation of our findings. Failure to show the effect of BFWP on wasting and underweight does not necessarily mean that there was no effect in the long run because of the complex nature of BFWP packages implementation. The workplace can be an ideal place to educate many adults, enhancing their understanding of nutrition and improving their health habits. Interventions at work focused on nutrition, physical activity, and health have shown positive results, including increased knowledge and confidence. This leads to improved habits such as eating more fruits and vegetables, reducing fat intake, avoiding alcohol and drugs, and establishing healthy eating routines [38].

The findings of this study can have significant implications for Ethiopian national nutrition policy and scaling up of WHO baby friendly workplace initiatives programs in the promotion of optimal children feeding practices as predictors of children’s nutritional status. Moreover. the findings indirectly highlight the importance of BFWP for the prevention of discontinuation of breast feeding in which public legislations of Ethiopia poorly protect the breastfeeding rights of mothers and the provided maternity leave period is also shorter than the recommended exclusive breastfeeding duration.

### Strength and limitations of the study

The strength of this study is the research is the first of its kind in Ethiopia to assess the effect of new initiatives of the baby-friendly workplace program on children’s nutritional status. However, we acknowledge the following limitations. The data were self-reported by the study participants,thus subject to recall and social desirability bias as the study relied on mothers’ memory.Therefore the information given could not be confirmed to be correct. Due to the small sample size, there was a lack of statistical significance is happen between BFWP initiatives and WHZ and WAZ scores, which limited our power to detect differences between the intervention comparable groups.

## Conclusion

Our study results are encouraging and seem to highlight that children aged 6–24 months who had not used baby friendly workplace initiatives were more stunted. The findings did not observe significant contributions of the BFWI on wasting and underweight.

Based on our study findings, we recommend that concerned stakeholders in the health sector focus on designing strategic efforts to enhance and scale up of BFWI programs. Further more, researchers should consider conducting a qualitative study to gain more insights in the contextual barriers, provider opinions, arrangements of BFWPI and health care workers perspectives on the implementation of BFWI. Finally,it is important to conduct longitudinal study which addresses the sustainability and potential long term effect of the program for a better policy implication and scaling up.

## Data Availability

The datasets used and/or analysed during the current study are available from the corresponding author based on reasonable request.

## Abbreviations

BFWPI: Baby Friendly Workplace initiatives
HAZ: Height for Age
WHZ: Weight for Height Z score
WFA: Weight for Age
